# Thirty-Two Case Reports of Synchronous Hematological Malignancy and Solid Tumor

**DOI:** 10.4274/tjh.galenos.2019.2019.0071

**Published:** 2019-11-18

**Authors:** Sha Liu, Xudong Wei, Yuanyuan Xiong, Ruihua Mi, Qingsong Yin

**Affiliations:** 1 The Second Affiliated Hospital of Zhengzhou University, Department of Hematology, Zhengzhou, P.R. China; 2 The Affiliated Cancer Hospital of Zhengzhou University, Department of Hematology, Zhengzhou, P.R. China

**Keywords:** Synchronous multiple primary cancer, Hematological malignancy, Solid tumor

## To the Editor,

Synchronous multiple primary cancer (SMPC) is defined as two or more malignancies diagnosed within 6 months of each other [1]. Its incidence is low, while the simultaneous occurrence of a hematological malignancy and a solid tumor is even less common with only cases reports provided [2,3,4,5,6]. We analyzed 32 patients with a synchronous hematologic malignancy and solid tumor at The Affiliated Cancer Hospital of Zhengzhou University from June 2012 to June 2018.

Patients and disease characteristics are shown in Table 1. These 32 patients included 17 males and 15 females. The median age at diagnosis was 58.5 years (range: 30-81 years). The incidence of SMPC in our center was approximately 0.05%, while this rate was reported as 0.5% in the literature [5]. The difference in this incidence might be attributable to differences in geography, environment, race, or various diagnostic criteria or, more importantly, the experience of the clinicians or the examination methods between studies.

The median interval between the diagnoses of these 2 primary malignancy types was 0.2 months (range: 0-5.3 months). Of the 32 cases, 2 patients were lost to follow-up while the other 30 patients completed the treatment: 3 cases with complete remission (CR), 9 cases with stable disease (SD), recurrence of gastric cancer in 1 case, 1 case of lymphoma recurrence, and 16 cases of death. The median overall survival (OS) of the 32 patients was 17.7 months (range: 1.3-68 months). Among the 16 deceased patients, there were 8 patients with a median age of 60.5 years (range: 44-78 years) who survived less than 10 months, and 4 of them had reported a family history of cancer. Eight patients were diagnosed with hematologic malignancies or solid tumors of stage III or IV. Among these 8 patients, 3 patients died early after surgery, 3 patients died of pulmonary infection after radiotherapy and chemotherapy, and 2 patients died of primary disease progression.

The pathogenesis of SMPC is not completely clear. Tabor et al.[7] found that tumors of different types and different tissues might originate from identical precancerous lesions. An Argentine study group found that 32% of multiple primary cancer patients reported a family history of cancer [8]. Genetic instability may play an important role in the development of multiple primary cancers. Based on the detection of replication errors on microsatellite loci, Horii et al. [9] found that genetic defects in the mismatch repair system represent a high-risk factor for multiple primary cancer patients. We identified 8 patients whose first-degree relatives had experienced malignant tumors in our study.

No standard treatment options are available for synchronous hematological malignancies and solid tumors. The degree of malignancy of each tumor, the response of each tumor to therapy, the therapy indications, and the general condition of the patient should be considered simultaneously. For patients who were diagnosed with a solid tumor and indolent lymphoma such as mucosa-associated lymphoid tissue lymphoma or marginal zone lymphoma, chemotherapy or I-131 radiotherapy was performed first to treat the solid tumor. However, for patients who were diagnosed with an early-stage solid tumor and highly aggressive lymphoma such as diffuse large B-cell lymphoma or anaplastic large-cell lymphoma, after surgical removal of the solid tumor, chemotherapy and sequential hematopoietic stem cell transplantation were administered to treat the lymphoma and at the same time regular postoperative follow-up for the solid tumor was performed.

## Figures and Tables

**Table 1:**
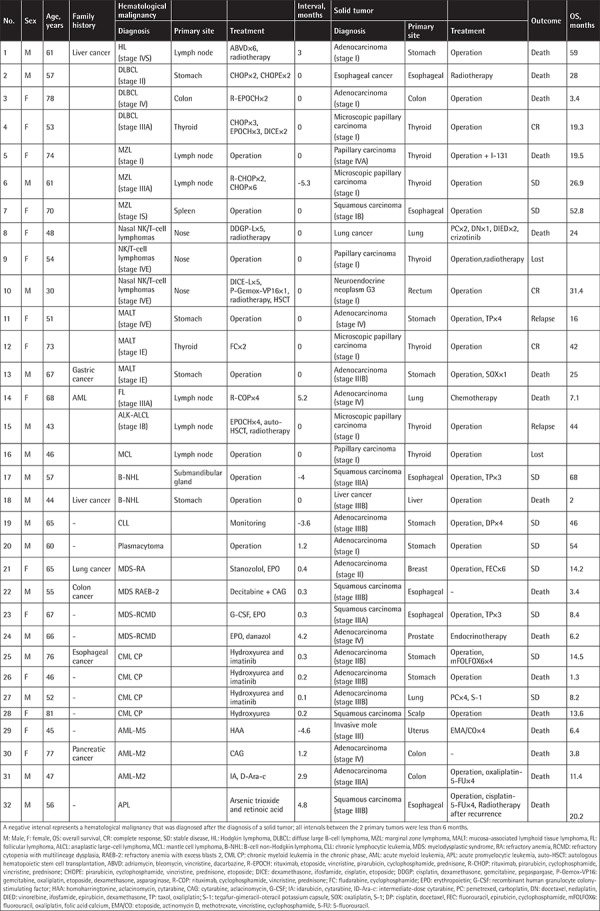
Clinical characteristics of 32 synchronous multiple primary cancer patients.
